# Maternal and neonatal factors associated with perinatal deaths in a South African healthcare institution

**DOI:** 10.4102/hsag.v25i0.1332

**Published:** 2020-12-07

**Authors:** Nthabiseng S. Malinga, Antoinette du Preez, Tinda Rabie

**Affiliations:** 1 NuMIQ Research Focus Area, School of Nursing Science, North-West University, Potchefstroom, South Africa

**Keywords:** neonates, neonatal deaths, perinatal deaths, stillbirths, maternity care

## Abstract

**Background:**

Research indicated the prevalence of perinatal deaths of infants immediately or up to a week after birth and includes fresh and macerated stillbirths and neonatal deaths. Worldwide, there is a decline in perinatal deaths. However, in South Africa, it is not the case. Often the quality of maternity care is considered as the most important contributing factor for these deaths. However, maternal and neonatal factors can also contribute.

**Aim:**

The aim of the study was to determine the maternal and neonatal factors associated with perinatal deaths in a single selected district hospital within the Free State Province of South Africa.

**Setting:**

The maternity unit of the largest district hospital in the specific district in the Free State Province of South Africa.

**Method:**

A clinical audit design was used. Units of analysis comprised the Perinatal Problem Identification Programme (PPIP) database of neonates born during 2015, and their mothers. A random sample of 384 alive neonates and an all-inclusive sample of 43 deceased neonates were taken from a total of 2319. Descriptive statistics were reported and Cohen’s effect sizes, *d*, were calculated to identify practically significant differences between the neonates in the alive and the deceased group, respectively.

**Results:**

Cohen’s effect sizes and logistical regression analyses indicate that the Apgar score recorded 10 min after birth, gestational age, birth weight of neonate and the parity of the mother were the most practically significant factors influencing a neonate’s chances of survival.

**Conclusion:**

Quality maternity care is not the only cause of perinatal mortality rates; maternal and neonatal factors are also contributors.

## Introduction

Perinatal mortality refers to the stillbirth or neonatal death of an infant immediately and up to 1 week after birth. Perinatal deaths are categorised as antepartum stillbirths – foetal deaths before the onset of labour, intrapartum stillbirths – foetal deaths before birth, and neonatal deaths, in other words, during the first 28 days (Smith 2016). Neonatal deaths are categorised in terms of early deaths, occurring within 5 days, or late deaths that take place within 28 days after birth. The present study focused on stillbirths and early neonatal deaths. Stillbirths are classified as either macerated or fresh (World Health Organisation [WHO] 2016). A fresh stillbirth implies that death occurred within 8 to 24 h prior to delivery (Tesfalul et al. 2020), whereas in a macerated stillbirth, the foetus had been retained for 6–12 h before birth (Mengesha & Dangisso 2020).

According to Allanson, Muller and Pattinson (2015), the global perinatal mortality estimates are 3 million stillbirths and 3 million neonatal deaths, many of which may have been prevented by optimal care. Nevertheless, worldwide, limited progress has been made to reduce neonatal deaths. The prevention of perinatal deaths, however, requires more accurate data in the international and South African public health agendas. Worldwide, in 2014, as many as 62 countries indicated a two-third reduction in the neonatal mortality rate, whereas South Africa showed an increase during the same period. This analysis was according to the United Nations International Children Emergency Fund (UNICEF 2014). Since 2009, South Africa’s National Department of Health (NDoH) set the target of a neonatal mortality rate of 14 deaths per 1000 live births (Mabaso, Ndaba & Mkhize-Kwitshana 2014). However, during 2013, the perinatal mortality rate in South Africa was more than double the target rate at 33.4 deaths per 1000 live births (Statistics SA 2015). Even after 2 years, the participating hospital did not reach the targeted reduction in neonatal mortality rate, considering the deaths being at the rate of 16.28 per 1000 live births.

### Problem statement

There is a widespread acknowledgement for the need to improve the quality and quantity of information regarding maternal mortality and perinatal deaths. However, there is a slow movement towards capturing and reviewing maternal and neonatal factors contributing to perinatal deaths, in order to make changes in practice (Kerber et al. 2015). The Perinatal Problem Identification Program (PPIP) database was developed to identify factors contributing to perinatal deaths to improve the quality of care. However, the raw data on the PPIP database did not supply sufficient information on the factors contributing to perinatal deaths in the particular district hospital where this study was conducted. Therefore, the present study focused on determining the practical significance between the different maternal and neonatal factors associated with perinatal deaths.

### Aim and objectives

The aim of this study was to determine maternal and neonatal factors associated with perinatal deaths. This aim was unpacked into the following objectives:

Objective 1: Identify and describe the demographic profile of the mothers and neonates.

Objective 2: Determine whether there are significant differences in the age, gravida, parity and health-risk- factors count between mothers with alive neonates and those whose neonates had died within 1 week after birth.

Objective 3: Ascertain whether the gender of the baby had a practical significant indicator to be born alive or dead and if there are practically significant differences between the birth weight, gestational age and Apgar scores of neonates who were born alive and those who had died within 1 week after birth.

## Research methods and design

### Study design

This study adopted a clinical audit design (Johnston et al. 2000; Swanepoel et al. 2015), which focused on a database without direct participation.

### Setting

The research setting was the data used through the PPIP database (Statistics SA 2015) of the maternity unit within the largest district hospital in the specific district in the Free State Province of South Africa.

### Unit of analyses and sampling strategy

The unit of analyses was the PPIP database of neonates born during 2015 and their mothers in the largest district hospital of the Free State Province. The PPIP database, was developed for the NDOH as a facility audit tool for perinatal deaths and contains information about maternal and perinatal states, which is transferred anonymously from maternity records (Rhoda et al. 2014). From the 2319 neonates born during 2015, random sampling was done of the alive (*n* = 384) and all- inclusive sampling of the deceased ones (*n* = 43). In the unit of analysis there were six sets of twins. The statistician recommended that only one infant of each set of twins should be included, because statistically every data point must be independent from other points. Naturally, twins are not independent, considering that they share the same mother with identical maternal health problems.

### Data collection

Data were collected by one of the authors of the study. The ex post facto data were extracted retrospectively and recorded on a specially designed Excel data extraction sheet, describing pre-selected maternal and neonatal factors associated with perinatal deaths. The Excel data sheet was developed by the authors with the assistance of a statistician as the PPIP database was not provided in an electronically usable format for data analysis.

The mentioned selected maternal variables were age, gravida, parity and maternal-risk-factor count, with the latter including aspects such as diabetes mellitus, syphilis, hypertension, HIV-positive, postpartum haemorrhage, ruptured uterus, placenta abruption, placenta praevia or prolonged and obstructed labour.

The selected neonatal variables were gender, birth weight, gestational age and Apgar score.

The data extraction sheet was pre-tested. The statistician selected 30 numbers randomly from the PPIP data base and the pre-selected factors were captured on the Excel data sheet. This was done to determine whether the study was feasible and appropriate and to detect possible flaws in the extraction sheet prior to data collection (Allanson et al. 2015). After analysing the pre-test, the statistician advised that numbered labels should be assigned to the captured data, with number 1 designating a male and number 2 a female neonate.

### Data analysis

Descriptive statistics were used to compile demographic profiles of the study’s sample. Cohen’s effect sizes were computed to determine the practical effect of differences between the means of the two strata on continuous variables for the mothers (e.g. parity, age, gravida) and the neonates (e.g. Apgar score, gestational age, weight). Phi coefficients were calculated for categorical variables (e.g. gender, mothers’ HIV status). A logistic regression analysis was done to identify the most practically significant predictors for neonates’ survival chances. The Statistical Analysis System [SAS] 2 computer 016 programme was used to analyse the data. Considering that the study sample was not a representative random one, inferential statistics were reported for the sake of completeness, but interpretations were based on Cohen’s effect sizes (Cohen 1988).

### Ethical considerations

Approval for this study was granted by the North-West University’s Ethics Committee (NWU-00357-16-SI), as well as the Head of the DoH of the Free State Province and the Chief Executive Officer of the participating hospital. Information from the 2015 PPIP database was used and no person participated directly in the study, as it was a clinical audit using ex post facto data.

## Results

The following section discusses the demographic profiles of the entire population of mothers and neonates and thereafter focuses on both deceased neonates as such and their mothers, retrospectively.

### Demographic profiles

#### Mothers

Out of the 384 mothers, 98.44% (*n* = 378) were single and 1.56% (*n* = 6) had twin pregnancies. Most of the women (80.74%; *n* = 310) were in the age group of 19 to 35 and 69.26% (*n* = 226) had been pregnant more than once. Out of the 384 recorded births, 37.50% (*n* = 144) of the women were primigravidas; 60.15% (*n* = 231) had given birth to 2 to 4 alive neonates; and 2.35% (*n* = 9) had given birth to 5 or more alive neonates. Being HIV-positive was the most common maternal-risk-factor count (36.72%; *n* = 141), whilst prolonged labour was the most common birth-related complication (2.60%; *n* = 10). Most women (96.34%; *n* = 369) came from urban areas and had normal vaginal deliveries (72.92%; *n* = 280) (see Table 1).

**TABLE 1:** Demographic profile of the mothers.

Categories	*N*	Frequency	Percentage	Mean (M)	Standard deviation (SD)
**Type of pregnancy**					
Singleton pregnancy	384	378	98.44	-	-
Twins	384	6	1.56	-	-
Triplets	384	0.00	0.00	0.00	0.00
**Age of mother**					
High-risk age (teenagers)	384	37	10.41	-	-
Low-risk childbearing age	384	310	80.74	-	-
High-risk age (advanced maternal age)	384	37	9.62	-	-
**Gravida**					
Primi gravida (first pregnancy)	384	118	30.73	-	-
Normal (2–4 pregnancies)	384	252	65.62	-	-
Multi-gravida	384	14	3.64	-	-
**Parity**					
Primi parity (first delivery)	384	144	37.50	-	-
Normal (2–4 deliveries)	384	231	60.15	-	-
Multi-parity (5+ deliveries)	384	9	2.34	-	-
**High risk factors**					
Diabetes	384	19	4.95	-	-
Syphilis	384	14	3.65	-	-
Hypertension	384	6	1.56	-	-
HIV	384	141	36.72	-	-
Post-partum haemorrhage	384	1	0.26	-	-
Ruptured uterus	384	3	0.78	-	-
Placenta abruption	384	1	0.26	-	-
Prolonged obstructed labour	384	10	2.60	-	-
None	384	189	49.22		
**Area of birth**				-	-
Urban	384	369	96.34	-	-
Rural	384	14	3.66	-	-
**Type of delivery**					
Normal vaginal delivery	384	280	72.92	-	-
Caesarean delivery	384	104	27.08	-	-

HIV, human immunodeficiency virus.

#### Neonates

Out of the 384 neonates, 48.70% (*n* = 187) were males and 51.30% (*n* = 197) were females; 34 (8.85%) were stillbirths and nine (2.34%) were neonatal deaths. Most of the neonates (64.58%; *n* = 248) were born full term at 38–40 weeks’ gestation. The average weight of the neonates was 3028.10 g (SD ± 607.63), the mean Apgar score after 5 min was 7.61 (SD ± 2.73) and the average Apgar score after 10 min was 8.74 (SD ± 3.02) (see Table 2).

**TABLE 2:** Demographic profile of the neonates.

Categories	*N*	Frequency	Percentage	Mean (M)	Standard deviation (SD)
**Gender**					
Male	384	187	48.70	-	-
Female	384	197	51.30	-	-
**Weight**	384	-	-	3028.10	-
**Apgar score**					
5 min	384	-	-	7.61	2.74
10 min	384	-	-	8.74	3.02
**Gestational age**					
Preterm neonate	384	129	33.59	-	-
Full-term neonate	384	248	64.58	-	-
Post-maturity	384	7	1.83	-	-
**Type of death**					
Stillbirth	384	34	8.85	-	-
Neonatal birth	384	9	2.34	-	-
**Outcome of birth**					
Alive	384	341	88.80	-	-
Dead	384	43	11.20	-	-

#### Deceased neonates’ mothers

Of the neonates born, more singleton neonates died (95.35%; *n* = 41) than twins (4.65%; *n* = 2). A quarter (25.6%; *n* = 11) of the deceased neonates’ mothers fell in the maternal high-risk age groups of teenagers (14–18 years) and advanced age (36–44 years). From the perinatal deaths, 60.09% (*n* = 26) were multi-gravidas. Nearly two-thirds (65.12%; *n* = 28) were multi-parity. The largest percentage (37.21%; *n* = 16) tested HIV-positive, whilst no mother suffered from hypertension, postpartum haemorrhage or presented with placenta praevia. Prolonged/obstructed labour occurred twice (4.65%; *n* = 2) and most mothers (69.77%; *n* = 30) had normal vaginal deliveries. The detail for the deceased neonates’ mothers is presented in Table 3.

**TABLE 3:** Demographics of deceased neonates’ mothers (*n* = 43).

Categories	Frequency	Percentage
**Mothers**		
**Type of pregnancy**		
Singleton	41	95.35
Twins	2	4.65
**Age of mother**		
High-risk age (teenagers aged 14–18)	6	13.95
Low-risk age (normal childbearing age 19–35 years)	32	74.44
High-risk age (advanced age 36–44 years)	5	11.65
**Gravida**		
Primi gravida	17	39.53
Multi-gravida	26	60.09
**Parity**		
Primi parity	15	34.88
Multi-parity	28	65.12
**Health risk factors**		
Diabetes mellitus	4	9.30
Syphilis	1	2.32
Hypertension	0	0.00
HIV-positive	16	37.21
Postpartum haemorrhage	0	0.00
Ruptured uterus	1	2.33
Placenta abruption	1	2.33
Placenta praevia	0	0.00
Prolonged/obstructed labour	2	4.65
Other	18	41.86
**Type of delivery**		
Normal vaginal delivery	30	69.77
Caesarean sections	13	30.23

HIV, human immunodeficiency virus.

#### Deceased neonates

The demographic profile of the deceased neonates indicates that there was almost the same number of deceased males and females: 48.84% (*n* = 21) and 51.16% (*n* = 22), respectively. According to the gestational age of the neonates, 58.15% (*n* = 25) were preterm; 37.21% (*n* = 16) were full-term; and 4.66% (*n* = 2) were post mature. The average weight of deceased neonates was 2508.91 g (SD ± 1011.03), indicating that the lower the birth weight, the higher the chances of perinatal deaths. The mean Apgar score of the deceased neonates at 5 min was 0.83 (SD ± 1.85) and the mean after 10 min was 0.69 (SD ± 2.25), which indicates that lower Apgar scores imply a higher mortality risk for neonates.

Table 4 portrays the descriptive statistics and Cohen’s effect sizes for continuous variables related to mothers and neonates, indicating differences on the status of the baby.

**TABLE 4:** Cohen’s effect sizes related to mothers and dead versus alive neonates.

Group	*N*	M	SD	*p* (when random sampling is assumed)	*d*
**Mothers**					
**Age**					
Dead neonates	43	25.40	6.31	0.30	0.17
Alive neonates	341	26.40	6.23		
**Gravida**					
Dead neonates	43	2.12	1.21	0.37	0.14
Alive neonates	341	2.28	1.20		
**Parity**					
Dead neonates	43	1.16	1.11	≤ 0.01†	0.86§
Alive neonates	341	2.12	1.12		
**Health risk factors**					
Dead neonates	43	0.58	0.70	0.46	0.12
Alive neonates	341	0.50	0.60		
**Neonates**					
**Birth weight**					
Dead neonates	43	2508.9	1011.0	≤ 0.01†	0.58‡
Alive neonates	341	3093	501.3		
**Apgar after 5 min**					
Dead neonates	43	0.84	1.85	≤ 0.01†	4.12§
Alive neonates	341	9.75	0.64		
**Apgar after 10 min**					
Dead neonates	43	0.70	2.25	≤ 0.01†	4.02§
Alive neonates	341	9.75	0.64		
**Gestational Age**					
Dead neonates	43	35.26	4.26	≤ 0.01†	0.60Δ
Alive neonates	341	37.82	1.93		

† , Statistically significant at 0.01 level according to *t*-test results for independent groups;

‡ , Medium effect in practice;

§ , Large and also practically significant.

SD, standard deviation. Guideline for Cohen’s effect sizes are as follows: *d* = |0.2| small effect, *d* = |0.5| medium effect and noticeable with the naked eye, *d* > = |0.8| large effect (Cohen 1988).

When calculating practically significant effects, it was found that age and gravida as well as health risk factors of the mothers did not show any practically significant effect on the survival status of the neonate (*d* < 0.2). However, mothers of alive neonates indicated a mean parity of 2.12, whilst the mothers of those who were deceased indicated a mean parity of 1.16, implying that the neonates’ survival chances improved as their mothers’ number of alive children increased. The mean birth weight (2508.9 g) of the deceased neonates differed from the mean birth weight (3093.0 g) of the alive ones with a medium effect size, implying that those who were deceased weigh less than those who are alive. The mean Apgar score at 5 min was 0.84 for deceased neonates and 9.75 for alive ones, and the *d* value was 4.12, thereby indicating a practically significant higher survival chance for neonates with higher Apgar scores. The mean Apgar score after 10 min for deceased neonates was found to be 0.70 and for alive neonates, 9.75 (*d* = 4.02). These results imply that the neonates who were alive had significantly higher Apgar scores at 10 min than the deceased ones. The mean gestational weeks for deceased neonates was found to be 35.26 weeks and for alive ones it was 37.82 weeks (*d* = 0.60), indicating a practical significance between deceased and alive neonates. From all neonatal variables, the Apgar score provided the strongest indication about the infant’s survival. The Phi coefficients of all categorical variables were calculated at less than 0.3 and thus showed no practical relationship with the neonates’ survival chances. From these results it can be inferred that health risk factors of the mothers and neonates’ gender had no practical effect.

The forward logistic regression indicated that the Apgar score at 10 min after birth and the mother’s parity predicted the infants’ survival status correctly in 98.7% of the cases. Table 5 presents strong predictions by the logistical regression model.

**TABLE 5:** Classification table to illustrate predictive power of the logistic regression model.

Variable	Predicted group		
	Dead	Alive	% Correct
**Actual group**			
Dead	39	4	90.7
Alive	1	340	99.7
**Overall correct**	**-**	**-**	**98.7**

## Discussion

As was pointed out previously, the findings showed no practically significant relationship between the survival status of the infant and maternal health risk factors of the mother or the neonates’ gender. However, parity was shown to have a practically significant impact on the neonates’ survival chances, thus implying that the higher the parity, the lower the possibility for perinatal deaths.

Neonatal factors (i.e. birth weight, gestational age and Apgar scores) were shown to have practical implications on these babies’ survival chances. Furthermore, findings from the present study showed that the deceased neonates weigh less at birth than the alive ones. A similar trend was reported by Agbozo, Abubakari and Der (2016), who investigated the prevalence of low birth weight, macrosomia and stillbirth and their relationship to associated maternal risk factors in Ghana.

In the present study, the mean of the weight of deceased neonates was found to be 519.2 g less than that of alive ones, indicating that the lower the gestational age, the higher the risk for neonatal deaths. This finding is in line with that of Amson et al. (2006), who found a similar trend and repeated that premature gestation and birth weight increase the chances of neonatal deaths. In addition, findings for the Apgar scores at 5 and 10 min after birth indicated that the higher the Apgar score, the higher the survival chances for the neonate. The Apgar score at 10 min after birth was the best indicator as to whether the neonate would survive or not.

### Limitations of the study

Despite the contributions of the findings to the field of study, certain limitations must be pointed out. Firstly, the findings represented only a single district hospital. Therefore, these findings may not be generalisable to other sites but could be used as a guideline for improving neonatal outcomes. Secondly, only data recorded on the PPIP system were utilised. Thus, the financial resources as well as the organisational or personnel factors of the hospital related to neonatal deaths were not considered. Finally, the PPIP data did not reflect the neonates’ survival status at 7 days of age but merely at the time of discharge from the hospital, which could be 6–8 h after a neonate’s birth.

### Recommendations for practice

Based on the findings, the following recommendations can be made for the midwifery practice.

If the parity of the pregnant mother is high, she must be monitored closely during antenatal, postnatal stages and birth, because the higher the female parity, the higher the chance of neonatal death.

Low birth weight is associated with higher risk of neonatal death and special attention should be paid to low birth weight and neonates who are small for their gestational age.

Future studies can include interviews with mothers of neonates to identify challenges which the pregnant women might encounter that might influence neonatal death rate in a specific area. Challenges may include cultural, social, financial and/or personal effects.

Interviews with midwives may identify service-related challenges that might contribute to neonatal deaths.

## Conclusion

According to the findings, maternal factors (age, gravida and health risk factors) indicated no practically significant effect on the status of the baby because these factors had *d* values of less than 0.2. However, parity played a practically significant role as the *d* value between the deceased and alive neonates was calculated at 0.86, thus the mother’s higher parity implied a better chance for the baby to live. Therefore, nulliparous women, especially at a very young age, also indicated higher rates of perinatal death.

Neonatal factors, with practical implications for the infants’ survival chances, include birth weight, gestational age and Apgar scores. The *d* value of 0.56 implies that the deceased neonates weigh less at birth than the alive ones. Therefore, the lower the birth weight, the stronger the chances for neonatal deaths. The *d* value of 0.60 for gestational age indicates that the lower the gestational age, the higher the risk for neonatal deaths; in other words, with a low gestational age, the chances are more for neonatal death. Apgar scores at both 5 (*d* = 4.12) and 10 min (*d* = 4.02) after birth provided the most significant indicators of the neonates’ survival chances. The higher the Apgar score, the higher the neonates’ survival chances. It is crucial to identify and address risks associated with a pregnant women’s parity and the neonates’ birth weight, gestational age and especially the Apgar score (5 and 10 min after birth). If such proactive measures can be implemented, certain neonates’ chances of survival could be improved.
